# Comparative Effects of Laparotomy and Laparoscopy‐Assisted Embryo Transfer on Oxidative Stress and Thiol–Disulphide Homeostasis in Tuj Ewes

**DOI:** 10.1002/vms3.71023

**Published:** 2026-06-12

**Authors:** Murat Can Demir, Merve Sena Demir, Mustafa Makav, Semra Kaya, Taygun Gökdemir, Nail Tekin Önder, Mushap Kuru, Cihan Kaçar

**Affiliations:** ^1^ Department of Obstetrics and Gynecology, Faculty of Veterinary Medicine Kafkas University Kars Türkiye; ^2^ Department of Physiology, Faculty of Veterinary Medicine Kafkas University Kars Türkiye; ^3^ Department of Reproduction and Artificial Insemination, Faculty of Veterinary Medicine Kafkas University Kars Türkiye

**Keywords:** embryo transfer, laparoscopy, laparotomy, oxidative stress, sheep, thiol–disulphide homeostasis

## Abstract

**Objectives:**

In this study, it was aimed to compare the effects of embryo transfer performed via laparotomy and laparoscopy on thiol–disulphide homeostasis and some oxidative stress markers in Tuj ewes.

**Methods:**

In the study, 10 Tuj ewes were randomly divided into two groups (laparotomy, *n* = 5; laparoscopy, *n* = 5). A progesterone‐based synchronization protocol was applied to all animals. Blood samples were collected immediately before surgery, immediately after surgery, and at 24 and 48 h postoperatively. Serum levels of glutathione (GSH), malondialdehyde (MDA), total thiol (TT), native thiol (NT), disulphide (Ds) and thiol–disulphide ratios were determined.

**Results:**

Group, time, and group × time interactions were found to be statistically significant for GSH, MDA, TT, and NT parameters (*p* < 0.05). In the laparotomy group, GSH, TT, and NT levels markedly decreased during the postoperative period (*p* < 0.05), whereas MDA levels showed a significant increase, particularly at 48 h (*p* < 0.05). In the laparoscopy group, changes in oxidative stress markers were found to be more limited. Although no significant differences were observed between the groups in terms of disulphide levels and thiol–disulphide ratios (*p* > 0.05), time‐dependent changes in these parameters were statistically significant (*p* < 0.05). In the laparotomy group, significant negative correlations were detected between MDA and antioxidant parameters (*p* < 0.05), while strong positive correlations were observed among thiol parameters (*p* < 0.01).

**Conclusions:**

As a result, it was concluded that the surgical approach used in embryo transfer affects the systemic oxidative stress response and that thiol–disulphide homeostasis can be considered a sensitive biomarker for revealing these differences. It was determined that the laparoscopic embryo transfer method disrupts redox balance to a lesser extent and better preserves the antioxidant defence system compared with laparotomy.

## Graphical abstract

Laparoscopic embryo transfer in Tuj ewes induces less oxidative stress and better preserves thiol–disulphide homeostasis than laparotomy. These findings indicate that minimally invasive approaches better maintain redox balance and antioxidant defence during embryo transfer procedures.

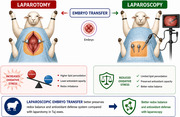

## 1 Introduction

Small ruminants play a crucial role in the economies and food production systems of developing countries. Their seasonally restricted reproductive activity has driven the development of assisted reproductive technologies aimed at increasing genetic gain and overcoming species‐specific reproductive limitations. Embryo transfer (ET) is one of the advanced reproductive biotechnologies applied in domestic animals and represents a powerful tool for genetic improvement in sheep and goats (Amiridis and Cseh 2012; Falchi et al. 2022).

In sheep and goats, ET can be performed using transcervical, laparotomic or laparoscopic approaches; however, due to anatomical constraints, ET procedures are most commonly carried out using laparotomy or laparoscopy (Cordeiro et al. 2003; Demir et al. 2025; Kershaw et al. 2005). Laparotomy has been widely used for many years; however, laparoscopy is increasingly preferred as a less invasive approach, owing to its association with limited tissue trauma, shorter recovery periods, and a lower risk of postoperative complications (Falchi et al. 2022; Kershaw et al. 2005).

Surgical interventions may cause oxidative stress by increasing the production of reactive oxygen species (ROS) through tissue damage and inflammatory responses (Agarwal et al. 2005; Halliwell 1994). While ROS at physiological levels are necessary for cellular signal transmission and normal reproductive functions, their excessive production leads to disruption of redox balance and causes oxidative damage to lipids, proteins, and nucleic acids (Halliwell 1994; Uttara et al. 2009). Both human and animal studies have demonstrated that oxidative stress can adversely affect ovarian function, luteal activity, embryo development and the maintenance of pregnancy (Agarwal et al. 2005; El Mouatassim 1999; Guerin et al. 2001). Pregnancy and the early embryonic period are physiologically sensitive processes in terms of oxidative stress due to increased metabolic requirements and intensive cellular activity (Garrel et al. 2010; Myatt and Cui 2004). Redox imbalances occurring during these stages have been reported to play a critical role in embryo viability and the continuation of pregnancy (Guerin et al. 2001; Rizzo et al. 2008).

Thiol–disulphide homeostasis is a dynamic and sensitive biomarker used in the evaluation of cellular redox balance. Thiol groups, which are one of the main components of intracellular antioxidant defence, are reversibly oxidized to disulphide (Ds) bonds under oxidative stress conditions, and changes occurring in this balance system provide important information about the presence and severity of oxidative stress (Biswas et al. 2006; Kohen and Nyska 2002). In recent years, thiol–disulphide homeostasis has been reported to be a reliable indicator in the evaluation of surgical stress, inflammation, and various pathological conditions (Circu and Aw 2010; Demir et al. 2026; Kundi et al. 2015).

In addition to thiol–disulphide balance, malondialdehyde (MDA), which is an end product of lipid peroxidation and glutathione (GSH), which is one of the main components of intracellular antioxidant defence, are biomarkers widely used in the evaluation of oxidative stress (Kohen and Nyska 2002; Ni̇sbet et al. 2008). The combined evaluation of these parameters allows a more comprehensive assessment of oxidative damage and antioxidant responses following surgical interventions (de Matos and Furnus 2000).

In the literature review conducted, no study has yet been found that comparatively examines the effects of ET procedures applied via laparotomy and laparoscopy on thiol–disulphide homeostasis together with classical oxidative stress markers in Tuj ewes. The aim of this study was to comparatively evaluate the effects of ET performed by laparotomy and laparoscopy on thiol–disulphide homeostasis as well as malondialdehyde and glutathione levels in Tuj ewes.

## 2 Materials and Methods

### 2.1 Ethical Approval

This study was approved by the Local Ethics Committee for Animal Experiments of Kafkas University (KAÜ–HADYEK‐2025‐125), and all procedures were conducted in accordance with national and international ethical guidelines for animal experimentation.

### 2.2 Animals

The study was conducted at the Education, Research and Application Farm of the Faculty of Veterinary Medicine, Kafkas University. A total of 10 clinically healthy Tuj ewes were included in the study. The animals were 3–4 years old, had at least one previous lambing, and had a body condition score ranging between 2.75 and 3.25 (1: extremely thin, 5: obese). Prior to the study, all animals underwent a general clinical examination, and individuals with any reproductive system pathology were excluded from the study.

### 2.3 Feeding

The ewes were housed under intensive management conditions and were provided with alfalfa and straw as roughage throughout the study period. In addition, each ewe received 450 g of concentrate feed per day containing 16% crude protein and 2700 kcal/kg metabolizable energy. Water was provided ad libitum during the study.

### 2.4 Experimental Design

The ewes were randomly allocated into two groups: a laparotomic ET group (*n* = 5) and a laparoscopic ET group (*n* = 5). For synchronization, all animals received an intravaginal medroxyprogesterone acetate sponge (60 mg, Esponjavet, Hipra, Spain) for 11 days. Twenty‐four hours before sponge removal, prostaglandin F_2_α (10 mg; Enzaprost‐T, Ceva, Türkiye) was administered, and pregnant mare serum gonadotropin (PMSG; 500 IU, Oviser, Hipra, Spain) was injected concurrently with sponge removal. Estrus detection was performed for two days, and ewes exhibiting estrus were identified. ET was carried out on Day 16 following sponge removal.

Frozen Tuj breed embryos, stored in liquid nitrogen at −196°C, were transferred to all recipients, and the uterine horn selected for transfer was determined based on the presence of the corpus luteum.

### 2.5 Surgical Procedures

To reduce potential risks during surgery, the ewes were fasted for 12 h prior to the operation. For premedication, atropine sulphate (0.02–0.05 mg/kg, s.c.; Vetaş Atropine 0.2%, Vetaş, Germany) was administered approximately 15 min before anaesthesia. Anaesthesia was induced using xylazine (0.1 mg/kg, i.m.; Rompun 2%, Bayer, Türkiye) and ketamine HCl (2.39 mg/kg, i.m.; Ketasol, Richter Pharma, Austria).

**FIGURE 1:**
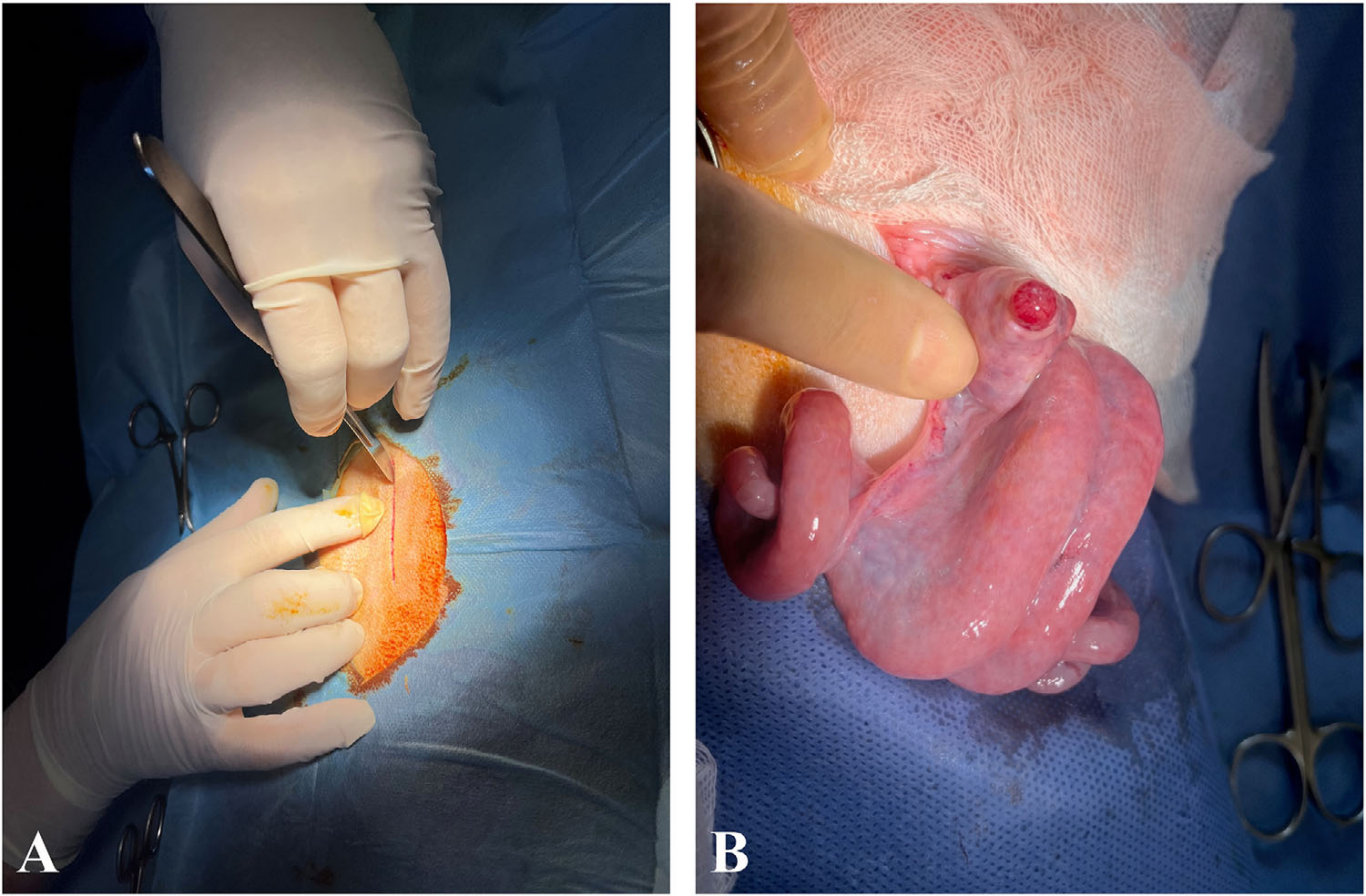
Laparotomic embryo transfer. (A) For laparotomic embryo transfer, access to the abdominal cavity was achieved through an abdominal incision. (B) Evaluation of the ovaries for the presence of follicles and corpus luteum.

*Laparotomic technique*: The abdominal region was shaved from the level of the umbilicus to the cranial aspect of the mammary lobes. A midline incision was made starting from the cranial part of the mammary lobes (Figure 1A), and the uterus and ovaries were exteriorized from the abdominal cavity. The ovaries were evaluated for the presence of follicles, cysts, corpus hemorrhagicum and corpus luteum (CL) (Figure 1B). During the transfer procedure, a small incision was made in the uterine horn using a 20‐gauge intravenous cannula, and the embryos were placed using a straw containing an appropriate transfer medium. After the uterus and ovaries were returned to their anatomical positions, the abdominal cavity was irrigated with at least 500 mL of 0.9% NaCl + heparin to prevent possible adhesions, and the incision line was closed using appropriate suture material.

**FIGURE 2:**
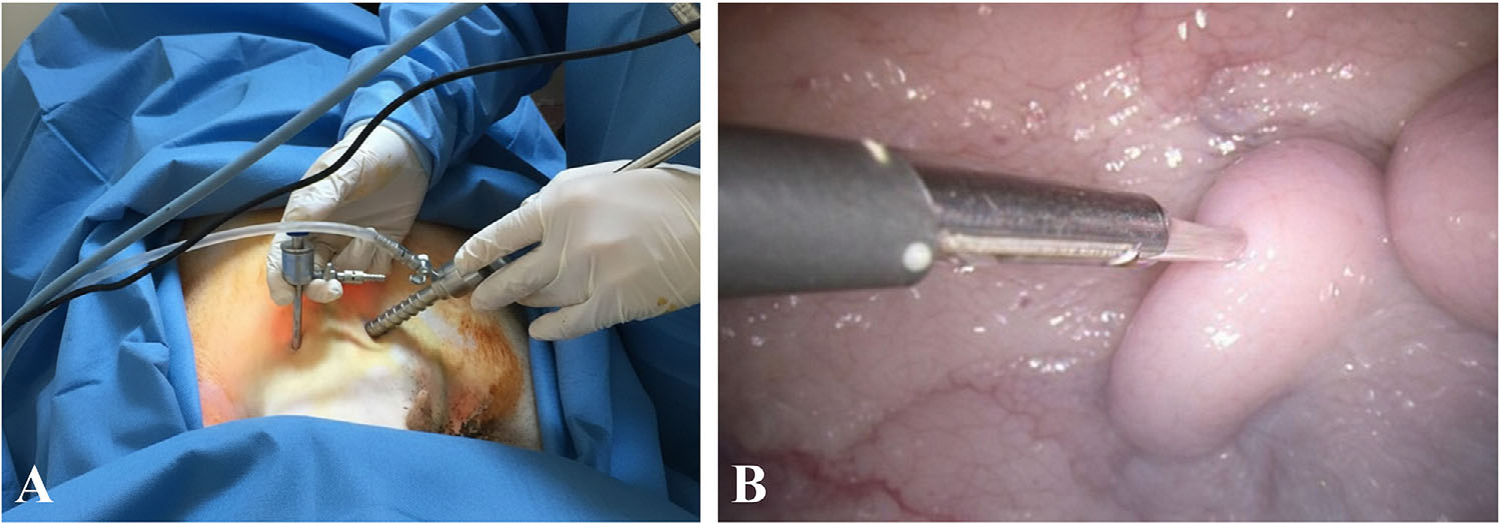
Laparoscopic embryo transfer. (A) Introduction of the laparoscope and auxiliary instruments into the abdominal cavity following transabdominal trocar placement. (B) Laparoscopic transfer of the embryo into the uterine horn containing the corpus luteum using a catheter.

*Laparoscopic technique*: Ewes undergoing laparoscopic ET were positioned in dorsal recumbency in the Trendelenburg position at an angle of approximately 40°. Two incisions were made approximately 10 cm cranial to the mammary gland and 2–3 cm lateral to the midline. Pneumoperitoneum was established by insufflation of CO_2_ using a Veress needle, after which a laparoscope was introduced through one incision and laparoscopic grasping forceps through the other (Figure 2A). The uterine horn containing the corpus luteum was grasped with forceps, and frozen ET was performed via a catheter inserted from the ventral aspect of the horn (Figure 2B). The incisions were closed using appropriate suture material.

Postoperatively, an antibiotic skin spray containing oxytetracycline hydrochloride (Neo‐Caf, Intervet/MSD, Italy) was applied to the surgical wound in all animals, and parenteral antibiotic treatment (oxytetracycline, 10 mg/kg, i.m.; Terramycin, Zoetis, Türkiye) was administered for 5 days.

### 2.6 Blood Sampling

Blood samples were collected from all ewes in both groups immediately before surgery, immediately after surgery, and at 24 and 48 h postoperatively. Blood was collected from the jugular vein using a holder and needle (BD Vacutainer, Becton, Dickinson and Company, USA) and transferred into 8.5 mL gel vacuum tubes (BD Vacutainer, Becton, Dickinson and Company, USA). Following collection, the tubes were centrifuged at 3000 rpm for 10 min at +4°C using an NF 400R centrifuge (Nüve, Türkiye). After centrifugation, serum samples were transferred into microcentrifuge tubes (Eppendorf AG, Hamburg, Germany) and stored at −20°C until further biochemical analyses.

### 2.7 Biochemical Analyses and Calculations

Glutathione (GSH) levels were determined using the method described by Ernest et al. (1963), while malondialdehyde (MDA) levels were measured according to the method of Yoshioka et al. (1979). Total thiol (TT) and native thiol (NT) levels were measured using the spectrophotometric method described by Erel and Neselioglu (2014).

Using TT and NT values, disulphide (Ds), Ds/TT × 100, Ds/NT × 100, and NT/TT × 100 ratios were calculated (Boğa Kuru and Makav 2024).

### 2.8 Statistical Analysis

The sample size was determined by an a priori power analysis using G*Power (version 3.1.9.7, Düsseldorf, Germany). For a repeated‐measures ANOVA with two groups and four time points, assuming a large effect size (*f* = 0.40), a correlation among repeated measures of 0.5, and an alpha error probability of 0.05, a total sample size of 10 ewes (*n* = 5 per group) was calculated to achieve a statistical power of 0.82.

Normality of continuous variables was assessed using the Shapiro–Wilk test. Data were presented as mean ± standard deviation. Two‐way analysis of variance was used to evaluate the effects of application technique (laparotomy and laparoscopy) and time (preoperative, postoperative, 24 h, and 48 h), as well as their interaction (time × group interaction), on oxidative stress parameters. When a significant interaction was detected, Tukey's multiple comparison test was applied to identify the source of differences between groups and time points. Pearson correlation analysis was performed to determine the direction and strength of the relationship between oxidative status (MDA and GSH) and thiol–disulphide homeostasis (total thiol, native thiol, disulphide). Correlation coefficients (*r*) and significance levels were calculated separately for the laparotomy group, the laparoscopy group and the total study population. Statistical analyses and data visualization were performed using GraphPad Prism (Version 10.0; GraphPad Software, Boston, MA, USA) and Python (Version 3.10; SciPy and Seaborn libraries). Statistical significance was set at *p* < 0.05.

## 3 Results

**FIGURE 3:**
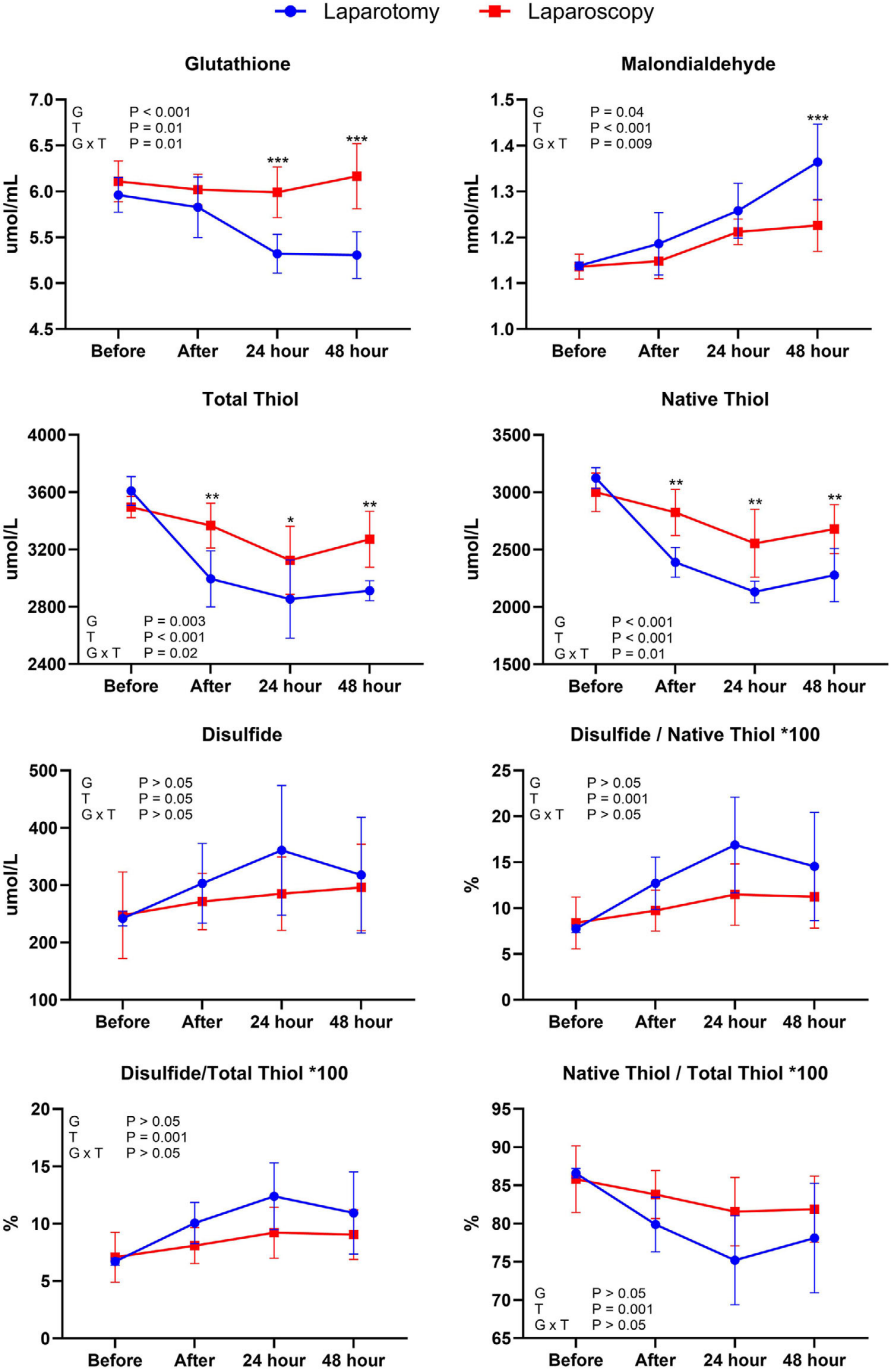
Time‐dependent changes in malondialdehyde, glutathione, and thiol–disulphide homeostasis parameters following laparotomic and laparoscopic embryo transfer. G: group effect (laparotomy vs. laparoscopy), T: time effect (before, immediately after, 24 h, and 48 h), G × T: interaction between group and time. *, **, *** indicate statistically significant differences between the laparotomic and laparoscopic groups on the respective sampling day (*p* < 0.05; *p* < 0.01; *p* < 0.001). The significant time effect (T) observed in all parameters was mainly driven by decreases in GSH, TT and NT levels at postoperative 24 h and 48 h compared with preoperative values, and by a progressive increase in MDA levels, particularly at 24 h and 48 h.

Time‐dependent changes and between‐group comparisons of GSH, MDA, and thiol–disulphide homeostasis parameters are presented in Figure 3.

GSH levels, statistically significant differences were detected between the laparotomy and laparoscopy groups at 24 and 48 h postoperatively (*p* < 0.001). According to repeated‐measures analysis, the effects of group (*p* < 0.001), time (*p* = 0.01), and the group × time interaction (*p* = 0.01) were found to be significant.

MDA levels, no statistically significant difference was observed between the groups at the preoperative period, immediately after surgery, or at 24 h postoperatively (P > 0.05). However, a statistically significant difference between the groups was detected at 48 h postoperatively (*p* < 0.001). In addition, the effects of group (*p* = 0.04), time (*p* < 0.001), and the group × time interaction (*p* = 0.009) were found to be significant.

TT levels, statistically significant differences between the groups were observed immediately after surgery (*p* < 0.01), at 24 h postoperatively (P < 0.05), and at 48 h postoperatively (*p* < 0.01). Repeated‐measures analysis revealed significant effects of group (*p* = 0.003), time (*p* < 0.001) and the group × time interaction (*p* = 0.02).

NT levels showed statistically significant differences between the groups immediately after surgery, at 24 h, and at 48 h postoperatively (all comparisons *p* < 0.05). The effects of group (*p* < 0.001), time (P < 0.001), and the group × time interaction (*p* = 0.01) were found to be significant.

With respect to Ds levels, no statistically significant differences were detected between the groups at any time point (*p* > 0.05). However, a statistically significant change was observed solely due to the effect of time (*p* = 0.05).

No significant differences between the groups were identified for the Ds/NT, Ds/TT, and NT/TT ratios. Statistically significant changes attributable only to the effect of time were observed for all these parameters (*p* = 0.001).

Post‐hoc comparisons of within‐group time‐dependent changes are presented in Table 1. In the laparotomy group, GSH levels significantly reduced at 24 and 48 h postoperatively compared with preoperative and immediate postoperative values (*p* < 0.05). MDA levels increased over time in both groups; however, this increase was more pronounced in the laparotomy group, with significantly higher values at 24 and 48 h postoperatively compared with baseline (*p* < 0.05). In the laparoscopy group, the time‐dependent increase in MDA levels was more limited and did not differ significantly between 24 and 48 h (*p* > 0.05). Total thiol (TT) levels in the laparotomy group were significantly decreased immediately after surgery, at 24 h, and at 48 h postoperatively (*p* < 0.01–0.05), whereas in the laparoscopy group, a significant reduction was observed only at 24 hours (P < 0.05), with levels at 48 hours not differing from baseline (*p* > 0.05). A similar pattern was observed for native thiol (NT) levels, which showed significant postoperative reductions at all time points in the laparotomy group (*p* < 0.05), while in the laparoscopy group, a significant decrease occurred only at 24 h (*p* < 0.05). Between‐group comparisons indicated that postoperative reductions in GSH, TT and NT levels were significantly greater in the laparotomy group than in the laparoscopy group at 24 and 48 h postoperatively (*p* < 0.05; Table 1).

**TABLE 1:** Time‐dependent changes in oxidative stress markers (GSH: µmol/L; MDA: Nmol/mL; TT: µmol/L; NT: µmol/L) in the laparotomy and laparoscopy groups.

Parameter	Group	Before	After	24 h	48 h
GSH (µmol/L)	Laparotomy	5.96 ± 0.19^a^	5.83 ± 0.33^a^	5.32 ± 0.21^b^	5.31 ± 0.26^b^
	Laparoscopy	6.11 ± 0.22	6.02 ± 0.17	5.99 ± 0.28	6.17 ± 0.35
MDA (nmol/mL)	Laparotomy	1.14 ± 0.01^a^	1.19 ± 0.07^a^	1.26 ± 0.06^b^	1.36 ± 0.08^c^
	Laparoscopy	1.14 ± 0.03^a^	1.15 ± 0.04^a^	1.21 ± 0.03^b^	1.23 ± 0.06^b^
TT (µmol/L)	Laparotomy	3608.33 ± 100.35^a^	2995.83 ± 195.92^b^	2854.17 ± 272.69^b^	2912.50 ± 69.10^b^
	Laparoscopy	3495.83 ± 73.95^a^	3366.67 ± 156.79^a^	3125.00 ± 237.32^b^	3270.83 ± 194.50^a^
NT (µmol/L)	Laparotomy	3124.32 ± 90.67^a^	2389.19 ± 129.22^b^	2132.43 ± 94.17^b^	2277.03 ± 231.46^b^
	Laparoscopy	3000.34 ± 167.40^a^	2823.65 ± 200.98^a^	2554.66 ± 296.24^b^	2678.38 ± 214.19^a^

*Note*: Data are presented as mean ± standard deviation (SD). Different superscript letters (a, b and c) within the same row indicate statistically significant differences between time points within the respective group (*p* < 0.05). Only parameters with a significant group × time interaction are included.

**FIGURE 4:**
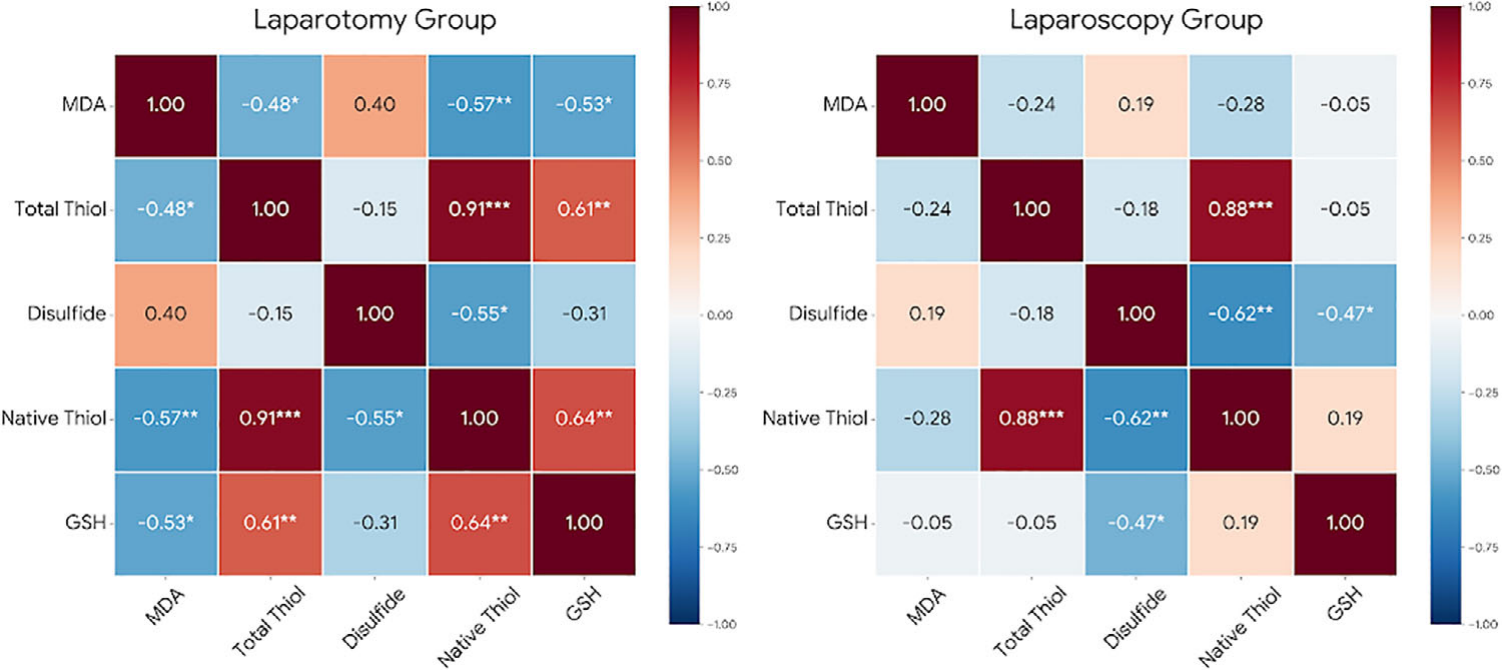
Correlations between oxidative stress markers and thiol–disulphide homeostasis parameters following laparotomic and laparoscopic embryo transfer.

Correlations between oxidative stress markers and thiol–disulphide homeostasis parameters were evaluated using correlation analysis, and the results are presented in Figure 4. In the laparotomy group, significant negative correlations were detected between MDA and TT, NT and GSH (*p* < 0.05), whereas strong positive correlations were observed between TT and NT and between TT and GSH (*p* < 0.01). In the laparoscopy group, a strong positive correlation was particularly identified between TT and NT (*p* < 0.01). When all animals were evaluated together, significant negative correlations were observed between MDA and TT, NT, and GSH (*p* < 0.01), while strong positive correlations were detected between TT and NT and between TT and GSH (*p* < 0.01).

## 4 Discussion

In this study, the effects of ET performed via laparotomy and laparoscopy on thiol–disulphide homeostasis and oxidative stress markers were compared in Tuj ewes, and the surgical approach resulted in distinct differences in the systemic oxidative response. The findings indicate that laparotomic group is associated with a more pronounced oxidative stress response and a greater disruption of redox balance compared with the laparoscopic technique. To the best of our knowledge, this study represents one of the first investigations to evaluate the effects of surgical techniques used for ET on oxidative stress responses by focusing on thiol–disulphide homeostasis.

Oxidative stress plays both regulatory and pathological roles at multiple stages of female reproductive physiology. ROS at physiological levels is involved in processes such as oocyte maturation, ovarian steroidogenesis, ovulation and implantation, whereas excessive ROS production is known to cause cellular damage and impair reproductive function (Agarwal et al. 2005; Jozwik 1999; Suzuki et al. 1999). It has previously been reported that surgical interventions may increase ROS production through tissue injury, inflammatory responses and metabolic stress (Halliwell 1994). In this context, evaluating the impact of surgical techniques used for ET on oxidative stress responses is of particular importance for the success of reproductive biotechnologies.

GSH is one of the principal components of cellular antioxidant defence and plays a critical role in maintaining redox balance. Increased GSH synthesis during oocyte maturation and early embryonic development, as well as its involvement in sustaining cellular redox homeostasis following fertilization, has been demonstrated in various species (Calvin et al. 1986; Miyamura 1995; Perreault et al. 1988; Yoshida et al. 1992). In our study, the significant decrease in GSH levels at 24 and 48 h postoperatively in the laparotomy group suggests that surgical trauma suppresses antioxidant defence capacity. The more stable GSH levels in the laparoscopy group support the idea that minimally invasive surgery keeps the systemic oxidative load more limited. These findings are consistent with previous studies that have shown a relationship between surgical stress level and antioxidant consumption (Agarwal et al. 2005).

MDA, as a reliable indicator of lipid peroxidation, reflects the extent of oxidative damage. Increased metabolic and uteroplacental circulatory requirements during pregnancy can lead to increased production of reactive oxygen species, resulting in a physiological oxidative stress response and increased MDA levels in early pregnancy (Csapo et al. 1972; Myatt 2006; Nielsen et al. 1997). In this study, the significant increase in MDA detected at 48 h postoperatively in the laparotomy group suggests an increase in lipid peroxidation due to acute surgical trauma, unlike the physiological oxidative response described in pregnancy. The more limited increase in MDA levels in the laparoscopy group supports the idea that surgical tissue damage and the accompanying inflammatory response occurred at a lower level.

Thiol–disulphide homeostasis represents a dynamic indicator of cellular redox status and is characterized by the conversion of thiol groups to disulphide bonds under oxidative stress conditions (Kohen and Nyska 2002). In the present study, although TT and NT levels decreased over time in both groups, this reduction was more pronounced in the laparotomy group. In parallel, higher disulphide ratios observed in the laparotomy group indicate a greater shift of redox balance toward an oxidative state. These findings suggest that thiol–disulphide homeostasis may serve as a sensitive and early marker of surgical stress when compared with classical oxidative stress indicators.

Disulphide levels did not show a significant change despite alterations in other oxidative stress markers, which may be attributed to the preferential oxidation of thiol groups beyond reversible disulphide formation under acute oxidative stress conditions. Under such conditions, thiols may undergo further irreversible oxidation to sulphinic and sulphonic derivatives, thereby limiting the accumulation of measurable disulphide bonds (Winterbourn and Hampton 2008). This is consistent with previous reports indicating that thiol depletion does not necessarily result in increased disulphide levels, particularly in conditions of intense oxidative stress (Erel and Neselioglu 2014).

Correlation analyses revealed a consistent pattern in the biochemical findings obtained in the study. Significant negative correlations between MDA levels and TT, NT and GSH in the laparotomy group reflect the balance between increased lipid peroxidation and decreased antioxidant reserves (Halliwell 1994). Strong positive correlations between TT and NT and GSH indicate that thiol‐based antioxidant systems operate within a holistic structure. The more limited correlation relationships in the laparoscopy group suggest that redox balance is more stably maintained in this group (Biswas et al. 2006; Erel and Neselioglu 2014). The correlation pattern observed when all animals are considered together supports a biologically consistent and significant relationship between thiol‐disulphide homeostasis parameters and oxidative stress markers. This suggests that thiol‐disulphide homeostasis can be used as a complementary tool in the holistic assessment of the surgical oxidative stress response.

## 5 Limitations

The present study has some limitations. First, the relatively small sample size (*n* = 5 per group) may limit the statistical power and generalizability of the findings. Second, only circulating oxidative stress markers and thiol–disulphide homeostasis parameters were evaluated. Although these systemic measurements are widely used to assess overall redox status, they may not fully reflect local tissue‐specific oxidative changes. Therefore, further studies including larger sample sizes and tissue‐level analyses are needed to better elucidate the local effects of different ET techniques. Third, reproductive outcome parameters such as pregnancy and conception rates were not evaluated in the present study. While our findings demonstrate the effects of laparotomic and laparoscopic ET techniques on systemic oxidative stress and thiol–disulphide homeostasis, the absence of reproductive performance data limits the interpretation of their biological and clinical relevance. Therefore, future studies that simultaneously evaluate embryo transfer outcomes together with oxidative stress markers will provide a more comprehensive perspective and help to better elucidate the relationship between different ET methods, redox balance, and reproductive efficiency in sheep. Fourth, although all preoperative and perioperative conditions were standardized, different anaesthetic protocols were applied due to the distinct procedural requirements of laparotomy and laparoscopy. Therefore, the potential influence of anaesthesia on oxidative stress parameters cannot be completely excluded. Future studies should consider the possible effects of different anaesthetic protocols on oxidative stress and design experimental groups accordingly.

## 6 Conclusion

This study supports the idea that the surgical approach used in embryo transfer is a determining factor in the systemic oxidative stress response, and that thiol–disulphide homeostasis can be used as a sensitive biomarker in revealing these differences. The findings revealed that laparoscopic embryo transfer disrupts redox balance less, better protects the antioxidant defence system, and results in more limited changes in oxidative stress markers. In embryo transfer applications in small ruminants, the laparoscopic method can be considered a more advantageous option, considering not only technical success but also biochemical and physiological responses and animal welfare.

## Author Contributions

**Murat Can Demir**: Conceptualization, Methodology, Software, Writing – Original Draft, Project administration. **Merve Sena Demir**: Data curation, Writing – Review & Editing, Software. **Mustafa Makav**: Visualization, Investigation. **Semra Kaya**: Software, Validation, Investigation. **Taygun Gökdemir**: Data curation, Software. **Nail Tekin Önder**: Validation, Investigation. **Mushap Kuru**: Formal analysis, Software, Data Curation. **Cihan Kaçar**: Methodology, Writing – Reviewing and Editing, Conceptualization.

## Funding

The authors have nothing to report.

## Conflicts of Interest

The authors declare no conflicts of interest.

## Ethical statement

The study was conducted in accordance with ethical principles following approval from the Kafkas University Animal Experiments Local Ethics Committee (KAÜ‐HADYEK‐2025‐125).

## Data Availability

The data that support the findings of this study are available from the corresponding author upon reasonable request.
